# Efficacy and Safety of Ensitrelvir for Asymptomatic or Mild COVID‐19: An Exploratory Analysis of a Multicenter, Randomized, Phase 2b/3 Clinical Trial

**DOI:** 10.1111/irv.13338

**Published:** 2024-06-18

**Authors:** Norio Ohmagari, Hiroshi Yotsuyanagi, Yohei Doi, Masaya Yamato, Takumi Imamura, Hiroki Sakaguchi, Hideki Yamanaka, Ryosuke Imaoka, Akimasa Fukushi, Genki Ichihashi, Takao Sanaki, Yuko Tsuge, Takeki Uehara, Hiroshi Mukae

**Affiliations:** ^1^ Disease Control and Prevention Center National Center for Global Health and Medicine Tokyo Japan; ^2^ The Institute of Medical Science The University of Tokyo Tokyo Japan; ^3^ Departments of Microbiology and Infectious Diseases Fujita Health University School of Medicine Toyoake Japan; ^4^ Division of Infectious Diseases University of Pittsburgh School of Medicine Pittsburgh Pennsylvania USA; ^5^ Department of General Medicine and Infectious Diseases Rinku General Medical Center Izumisano Japan; ^6^ Drug Development and Regulatory Science Division Shionogi & Co., Ltd Osaka Japan; ^7^ Research Division Shionogi & Co., Ltd Osaka Japan; ^8^ Department of Respiratory Medicine Nagasaki University Graduate School of Biomedical Sciences Nagasaki Japan

**Keywords:** asymptomatic infections, controlled clinical trial, COVID‐19 drug treatment, ensitrelvir, postexposure prophylaxis

## Abstract

**Background:**

This phase 2b/3, randomized, placebo‐controlled trial explored the efficacy and evaluated the safety of ensitrelvir. This trial involved individuals with asymptomatic infection with severe acute respiratory syndrome coronavirus 2 (SARS‐CoV‐2) and patients with mild symptoms of coronavirus disease 2019 (COVID‐19).

**Methods:**

The trial was conducted at 57 medical institutions in Japan, South Korea, and Vietnam (study period: January 6–August 14, 2022). Eligible participants were randomized (1:1:1) to the ensitrelvir 125‐mg, ensitrelvir 250‐mg, or placebo group, received the allocated intervention orally, and were followed up until Day 28. Participants self‐rated the severity of 14 typical COVID‐19 symptoms and recorded the data in an electronic diary.

**Results:**

In total, 572 participants (194, 189, and 189 in the ensitrelvir 125‐mg, ensitrelvir 250‐mg, and placebo groups, respectively) were included in the intention‐to‐treat population. Ensitrelvir 125‐mg group observed a 77% reduction in the risk of developing any of the 14 COVID‐19 symptoms or fever and a 29% reduction in the risk of worsening of such symptoms or fever versus placebo (statistically nonsignificant). The viral RNA, viral titer, and time to infectious viral clearance observed a statistically significant decrease versus placebo. Most treatment‐related adverse events (TEAEs) were mild to moderate in severity, and the most common TEAE observed across groups was a decrease in high‐density lipoprotein.

**Conclusions:**

Our exploratory results suggest a potential reduction in the risk of development or worsening of COVID‐19 symptoms with ensitrelvir. Ensitrelvir showed antiviral efficacy and was well tolerated.

**Trial Registration:** Japan Registry of Clinical Trials identifier: jRCT2031210350.

## 1 Introduction

The spread of severe acute respiratory syndrome coronavirus 2 (SARS‐CoV‐2) has posed a significant burden on global health security [1]. At least one‐third of the patients infected with SARS‐CoV‐2 remain asymptomatic and unaware of their infection [2]; however, shedding of infectious SARS‐CoV‐2 may persist for 10 days or longer in affected patients [3, 4], which could pose a potential risk of virus spreading to close contacts [4]. Indeed, the household secondary attack rate of the SARS‐CoV‐2 Omicron variant is estimated at 42.7% [5]. Antiviral treatment options that can prevent SARS‐CoV‐2 transmission may be useful in preventing the spread of infection.

Ensitrelvir is a novel SARS‐CoV‐2 3C–like protease inhibitor that has demonstrated efficacy in patients with mild‐to‐moderate coronavirus disease 2019 (COVID‐19) in clinical trial settings [6, 7, 8]. Here, we report an exploratory evaluation of the clinical and virologic efficacy and safety of ensitrelvir in individuals with asymptomatic SARS‐CoV‐2 infection and patients with mild COVID‐19 symptoms.

## 2 Materials and Methods

### 2.1 Study Design

This phase 2b/3 trial was conducted as part of a seamless, multicenter, randomized, double‐blind, placebo‐controlled, phase 2/3 study. Individuals with asymptomatic SARS‐CoV‐2 infection and patients with mild COVID‐19 symptoms were enrolled at 57 medical institutions in Japan, South Korea, and Vietnam (study period: January 6–August 14, 2022). Eligible participants were randomized (1:1:1) to the ensitrelvir 125‐mg, ensitrelvir 250‐mg, or placebo group, received the allocated intervention orally, and were followed up until Day 28.

This study was conducted in accordance with the principles of the Declaration of Helsinki, the Good Clinical Practice guidelines, and other applicable laws and regulations. The study protocol was approved by the institutional review boards of all participating institutions. Written informed consent was obtained from all participants or their legally acceptable representatives. This report follows the Consolidated Standards of Reporting Trials (CONSORT) reporting guideline for randomized studies.

### 2.2 Study Participants, Randomization, Blinding, and Treatment

Eligible participants were those aged 12 to < 70 years who tested positive by nucleic acid detection or antigen testing for SARS‐CoV‐2 within 120 h prior to randomization. Participants self‐assessed the severity of COVID‐19 symptoms using a questionnaire (Supplementary Methods) developed based on the US Food and Drug Administration guidance for assessing COVID‐19 symptoms in clinical trials [9]. Those who did not have a self‐rated COVID‐19 symptom score of 2 (*moderate*) or 3 (*severe*) among the predefined 12 COVID‐19 symptoms within 2 weeks before randomization were eligible for enrollment. Participants with an awake oxygen saturation of ≤ 93% (room air), who required supplemental oxygen, or who were likely to experience COVID‐19 exacerbation within 48 h of randomization were excluded. Further details regarding the inclusion and exclusion criteria are provided in the Supplementary Methods.

Randomization of participants was performed using an interactive response technology system and stratification by SARS‐CoV‐2 vaccination history (whether the first vaccination has been completed: yes vs. no). All participants and study staff were blinded to the treatment, and emergency unblinding per the investigator's request was allowed in case of adverse events to determine the appropriate therapy for the participant. Randomized participants received ensitrelvir (375 mg on Day 1 and 125 mg on Days 2 through 5, or 750 mg on Day 1 and 250 mg on Days 2 through 5) or matching placebo tablets, which were identical in appearance and packaging, without dose modification.

### 2.3 Outcomes and Assessments

Clinical efficacy was assessed based on the proportion of participants who experienced the development (asymptomatic individuals) or worsening (patients with mild COVID‐19 symptoms) of any of the 14 COVID‐19 symptoms or fever (Supplementary Methods) by Day 10 after treatment initiation. Additionally, SARS‐CoV‐2 viral RNA levels on Day 4, the proportion of participants with a positive SARS‐CoV‐2 viral titer, and the time to the first negative SARS‐CoV‐2 viral titer (infectious viral clearance) were evaluated for virologic efficacy. Participants self‐rated the severity of each COVID‐19 symptom, measured their body temperature using a thermometer, and recorded the data in a diary. SARS‐CoV‐2 viral titer and viral RNA were quantified using nasopharyngeal swabs (Supplementary Methods). Safety was assessed based on the incidence of adverse events. All safety data were evaluated by an independent data and safety‐monitoring board.

### 2.4 Statistical Analyses

This phase 2b/3 part was initially designed as a confirmatory trial but was changed to an exploratory evaluation during the study. The required sample size was calculated for the initially planned primary objective based on the published literature [2], which resulted in 143 participants required in each group. Assuming a dropout rate of approximately 10% due to a negative reverse transcription‐polymerase chain reaction result, enrollment of 480 participants (160 per group) was required (see Supplementary Methods for further details).

All statistical comparisons were performed at a two‐sided significance level of 0.05. All analyses were performed using SAS version 9.4 (SAS Institute Inc., Cary, NC, USA). Further details regarding the statistical analyses are provided in the Supplementary Methods.

## 3 Results

### 3.1 Participant Disposition and Baseline Characteristics

A total of 605 participants were randomized, of whom 572 (194, 189, and 189 in the ensitrelvir 125‐mg, ensitrelvir 250‐mg, and placebo groups, respectively) were included in the intention‐to‐treat (ITT) population (Figure S1). The mean (standard deviation [SD]) age in the ITT population was 37.9 (12.0), 40.9 (13.4), and 38.6 (13.0) years in the ensitrelvir 125‐mg, ensitrelvir 250‐mg, and placebo groups, respectively. Approximately 60% of participants were infected with SARS‐CoV‐2 Omicron subvariants, and most of the remaining participants had unidentified subvariants. Of the 572 participants, 70 (12.2%) were asymptomatic, 502 (87.8%) had mild symptoms, and 525 (91.8%) had a history of COVID‐19 vaccination (Table S1).

### 3.2 Clinical Efficacy

Overall, 4.3% and 20.0% of asymptomatic individuals in the ensitrelvir 125‐mg and 250‐mg groups, respectively, experienced the development of 14 COVID‐19 symptoms or fever (18.2% in the placebo group; risk ratio [95% confidence interval (CI)]: 0.23 [0.03–1.88] and 1.08 [0.31–3.73], respectively). Moreover, 17.4% and 20.5% of patients with mild symptoms in the ensitrelvir 125‐mg and 250‐mg groups, respectively, experienced worsening of 14 COVID‐19 symptoms or fever (23.9% in the placebo group; risk ratio [95% CI]: 0.71 [0.47–1.09] and 0.86 [0.57–1.28], respectively). These results were not statistically significant (Table 1). Among the five asymptomatic individuals who experienced symptom development in the ensitrelvir 250‐mg group, two met the definition of symptom development because of a transient elevation of body temperature with no accompanying symptoms. One participant was considered symptomatic because of transient shortness of breath.

**TABLE 1:** Proportion of participants experiencing the development or worsening of 14 COVID‐19 symptoms or fever by Day 10 (ITT population).

	Ensitrelvir 125 mg	Ensitrelvir 250 mg	Placebo
Development of 14 COVID‐19 symptoms or fever in asymptomatic individuals			
Participants with symptom development or fever, % (n/N)	4.3 (1/23)	20.0 (5/25)	18.2 (4/22)
*P*‐value (two‐sided, Mantel–Haenszel test)^a^	0.1293	0.9082	—
Risk ratio (95% CI)^a^	0.23 (0.03–1.88)	1.08 (0.31–3.73)	—
Worsening of 14 COVID‐19 symptoms or fever in patients with mild symptoms			
Participants with symptom worsening or fever, % (n/N)	17.4 (29/167)	20.5 (33/161)	23.9 (39/163)
*P*‐value (two‐sided, Mantel–Haenszel test)^a^	0.1210	0.4521	—
Risk ratio (95% CI)^a^	0.71 (0.47–1.09)	0.86 (0.57–1.28)	—

*Note:* The ITT population comprised all randomized participants who tested positive for SARS‐CoV‐2 infection at baseline, as confirmed by an RT‐PCR test based on the nasopharyngeal swab sample.

Abbreviations: CI, confidence interval; COVID‐19, coronavirus disease 2019; ITT, intention to treat; RT‐PCR, reverse transcription‐polymerase chain reaction; SARS‐CoV‐2, severe acute respiratory syndrome coronavirus 2.

^a^ The analyses included SARS‐CoV‐2 vaccination history (yes or no) as a stratification factor.

### 3.3 Virologic Efficacy

**FIGURE 1:**
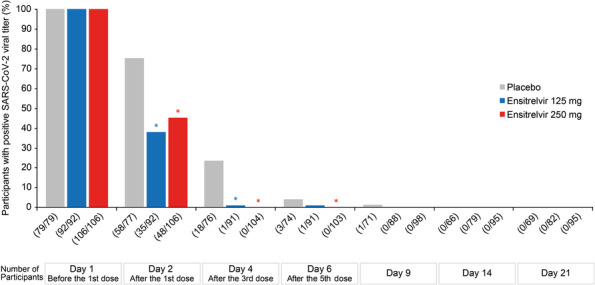
Proportion of participants with a positive SARS‐CoV‐2 viral titer (mITT population). The mITT population comprised all randomized participants who tested positive for SARS‐CoV‐2 infection and had detectable SARS‐CoV‐2 viral titers at baseline. A positive SARS‐CoV‐2 viral titer was defined as ≥ 0.75 log_10_TCID_50_/mL. *p < 0.05 vs. placebo using the Mantel–Haenszel test stratified by SARS‐CoV‐2 vaccination history (yes or no). mITT, modified intention to treat; SARS‐CoV‐2, severe acute respiratory syndrome coronavirus 2; TCID_50_, 50% tissue culture infectious dose.

The change from baseline in the SARS‐CoV‐2 viral RNA level was significantly lower in the ensitrelvir 125‐mg and 250‐mg groups than in the placebo group on Day 4 (*p* < 0.0001 for both) (Figure S2A,B). Similarly, the proportion of participants with a positive SARS‐CoV‐2 viral titer was significantly lower in the ensitrelvir 125‐mg and 250‐mg groups than in the placebo group on Days 2 and 4 (*p* < 0.0001 for all) (Figure 1). The time to first negative SARS‐CoV‐2 viral titer was significantly shorter in the ensitrelvir 125‐mg and 250‐mg groups than in the placebo group (*p* < 0.0001 for both) (Figure S3).

### 3.4 Safety

Treatment‐emergent adverse events (TEAEs) were reported in 88 (43.8%), 115 (56.9%), and 43 (21.4%) participants in the ensitrelvir 125‐mg, ensitrelvir 250‐mg, and placebo groups, respectively, in the safety analysis population; most TEAEs were mild to moderate in severity. The most common TEAE observed across groups was a decrease in high‐density lipoprotein, which occurred in 61 (30.3%), 91 (45.0%), and 4 (2.0%) participants in the ensitrelvir 125‐mg, ensitrelvir 250‐mg, and placebo groups, respectively. Two participants in the ensitrelvir 250‐mg group experienced serious TEAEs (diverticulitis and nausea, *n* = 1 each), but neither event was treatment related. No TEAEs leading to death were reported (Table S2).

## 4 Discussion

In this phase 2b/3 trial, ensitrelvir 125‐mg treatment observed a nonsignificant 77% reduction in the risk of developing 14 COVID‐19 symptoms or fever in asymptomatic individuals vs. placebo. Ensitrelvir treatment observed nonsignificant reductions in the risk of worsening of such symptoms or fever in patients with mild symptoms vs. placebo and statistically significant viral load reductions compared with placebo. Overall, there were no notable differences in efficacy or safety between the ensitrelvir 125‐mg and 250‐mg groups. These findings are consistent with the results from previous clinical trials of ensitrelvir for patients with mild‐to‐moderate COVID‐19 [6, 7, 8].

Published studies indicate that presymptomatic and asymptomatic individuals may be a source of SARS‐CoV‐2 transmission [10]. Moreover, evidence suggests an association between early antiviral therapy and a reduced risk of post‐COVID conditions (PCCs) [11]. Antiviral treatment for asymptomatic individuals to prevent SARS‐CoV‐2 transmission and PCCs may be helpful from a public health perspective. The reduction in viral RNA and the shorter time to a negative viral titer vs. placebo seen in the ensitrelvir groups may contribute to a shortened period of infection as well as a reduction in the risk of viral transmission and PCCs.

Our exploratory results suggest a potential reduction in the risk of development or worsening of COVID‐19 symptoms with ensitrelvir. The treatment showed antiviral efficacy in individuals with asymptomatic SARS‐CoV‐2 infection and patients with mild COVID‐19 symptoms. Ensitrelvir was well tolerated, and no new safety concerns were identified.

## Author Contributions

**Norio Ohmagari:** conceptualization, writing–review and editing, supervision. **Hiroshi Yotsuyanagi:** conceptualization, writing–review and editing. **Yohei Doi:** conceptualization, writing–review and editing. **Masaya Yamato:** conceptualization, writing–review and editing. **Takumi Imamura:** conceptualization, formal analysis, writing–review and editing, visualization. **Hiroki Sakaguchi:** conceptualization, formal analysis, writing–review and editing, visualization. **Hideki Yamanaka:** conceptualization, data curation, writing–review and editing. **Ryosuke Imaoka:** conceptualization, data curation, writing–review and editing. **Akimasa Fukushi:** conceptualization, data curation, writing–review and editing. **Genki Ichihashi:** conceptualization, data curation, writing–review and editing, project administration. **Takao Sanaki:** conceptualization, methodology, formal analysis, data curation, writing–review and editing. **Yuko Tsuge:** conceptualization, writing–original draft, supervision, project administration. **Takeki Uehara:** conceptualization, writing–review and editing, supervision, project administration. **Hiroshi Mukae:** conceptualization, data curation, writing–review and editing.

## Disclosure

Employees of the sponsor were involved in the study design; collection, analysis, and interpretation of data; writing of the report; and decision to submit the article for publication.

## Ethics Statement

This study was conducted in accordance with the principles of the Declaration of Helsinki, Good Clinical Practice guidelines, and other applicable laws and regulations. The study protocol was approved by the institutional review boards of all participating institutions.

## Consent

Written informed consent was obtained from all participants or their legally acceptable representatives.

## Conflicts of Interest

Hiroshi Yotsuyanagi has received honoraria for lectures from and chairs in sponsored symposiums for Shionogi and ViiV Healthcare, received travel and meeting support from Shionogi, and serves as an advisory board member of Shionogi and President of the Japanese Society of Infectious Diseases. Yohei Doi has received grants from Entasis and Shionogi; consulting fees from GSK, Moderna, Gilead Sciences, Shionogi, Fujifilm, Meiji Seika Pharma, Pfizer, and AbbVie; and lecture fees from Gilead and Shionogi. Masaya Yamato has received lecture fees from Shionogi. Takumi Imamura, Hiroki Sakaguchi, Hideki Yamanaka, Ryosuke Imaoka, Akimasa Fukushi, Genki Ichihashi, Takao Sanaki, Yuko Tsuge, and Takeki Uehara are full‐time employees of Shionogi & Co., Ltd. and may hold stocks in the company. Hiroshi Mukae has received support for the present manuscript from Shionogi; consulting fees from Shionogi and MSD; and lecture fees from Shionogi, MSD, Gilead Sciences, AstraZeneca, Pfizer, and GSK. Norio Ohmagari has no conflict of interest.

## Supporting information


**Table S1.** Baseline demographics and clinical characteristics (ITT population).
**Table S2.** Summary of adverse events (safety analysis population).
**Figure S1.** Participant flow.
**Figure S2.** (A) Observed values and (B) change from baseline in SARS‐CoV‐2 RNA levels (ITT population).
**Figure S3.** Kaplan–Meier plot for the time to first negative infectious SARS‐CoV‐2 viral titer (infectious viral clearance) (mITT population).

## Data Availability

Shionogi & Co., Ltd. is committed to disclosing the synopses and results of its clinical trials and sharing clinical trial data with researchers upon reasonable request. For further details, please refer to the websites of Shionogi & Co., Ltd. (https://www.shionogi.com/shionogi/global/en/company/policies/shionogi‐group‐clinical‐trial‐data‐transparency‐policy.html) and Vivli (https://vivli.org/).
