# Supplementary material for: Efficacy and Safety of Ensitrelvir for Asymptomatic or Mild COVID‐19: An Exploratory Analysis of a Multicenter, Randomized, Phase 2b/3 Clinical Trial
Source: Influenza Other Respir Viruses. 2024 Jun 18;18(6):e13338. doi: 10.1111/irv.13338 (PMC11187911; doi:10.1111/irv.13338)
Supplement: Supplementary file 1 — Table S1. Baseline demographics and clinical characteristics (ITT population). Table S2. Summary of adverse events (safety analysis population). Figure S1. Participant flow. Figure S2. (A) Observed values and (B) change from baseline in SARS‐CoV‐2 RNA levels (ITT population). Figure S3. Kaplan–Meier plot for the time to first negative infectious SARS‐CoV‐2 viral titer (infectious viral clearance) (mITT population). [file IRV-18-e13338-s001.docx]

**SUPPORTING INFORMATION**

**Supplementary methods**

***COVID-19 symptom score***

The severity of COVID-19 symptoms was assessed using a questionnaire developed based on the US Food and Drug Administration guidance for assessing COVID-19 symptoms in clinical trials [1]. The severity of each of the 14 COVID-19 symptoms was rated by study participants using the following scores:

| **Symptom** | **Category** | **Symptom score** |
| --- | --- | --- |
| 1. Stuffy or runny nose | Respiratory symptoms | None = 0  Mild = 1  Moderate = 2  Severe = 3 |
| 2. Sore throat |  |  |
| 3. Shortness of breath (difficulty breathing) |  |  |
| 4. Cough |  |  |
| 5. Low energy or tiredness | General symptoms |  |
| 6. Muscle or body aches |  |  |
| 7. Headache |  |  |
| 8. Chills or shivering |  |  |
| 9. Feeling hot or feverish |  |  |
| 10. Nausea (feeling like you want to throw up) | Gastrointestinal symptoms |  |
| 11. Vomiting (throwing up) |  |  |
| 12. Diarrhea (loose or watery stools) |  |  |
| 13. Sense of smell^a^ | – | Same as usual = 0  Less than usual = 1  No sense of smell/taste = 2 |
| 14. Sense of taste^a^ |  |  |

^a^Not included in the 12 COVID-19 symptom assessment at screening.

COVID-19, coronavirus disease 2019.

***Inclusion and exclusion criteria***

Eligible participants were those aged 12 to < 70 years who tested positive for severe acute respiratory syndrome coronavirus 2 (SARS-CoV-2) within 120 hours prior to randomization and who did not have a self-rated coronavirus disease 2019 (COVID-19) symptom score of 2 (*moderate*) or 3 (*severe*) among the 12 COVID-19 symptoms within 2 weeks before randomization (see “Assessments of COVID-19 symptoms and body temperature” below; symptoms present prior to COVID-19 onset were excluded). The applicable SARS-CoV-2 tests included nucleic acid detection or quantitative/qualitative antigen testing using nasopharyngeal or nasal swabs. The use of saliva samples was also allowed for nucleic acid detection and quantitative antigen tests. To avoid excessive drug exposure, participants aged < 18 years should have recorded a body weight of ≥ 40 kg at enrollment. Women were eligible to participate if they were not pregnant, possibly pregnant, or breastfeeding.

The key exclusion criteria included the following: an awake oxygen saturation of ≤ 93% (room air); supplemental oxygen requirement; anticipated COVID-19 exacerbation within 48 hours of randomization in the opinion of the investigator; suspected active and systemic infections other than COVID-19 requiring treatment; current or chronic history of moderate or severe liver disease, known hepatic or biliary abnormalities (except for Gilbert’s syndrome or asymptomatic gallstones), or moderate-to-severe kidney disease; and blood donation. Participants who had used drugs for SARS-CoV-2 infection within 7 days prior to randomization, a strong cytochrome P450, family 3, subfamily A (CYP3A) inhibitor or inducer within 14 days prior to randomization, or St. John’s wort products within 14 days prior to randomization were also excluded.

***Assessments of COVID-19 symptoms and body temperature***

The severity of each of the 14 COVID-19 symptoms was self-rated using the symptom scores shown above on Day 1 (pre- and post-dose), twice daily (morning and evening) on Days 2 to 9, and once daily (evening) from Days 10 to 21 and recorded in an electronic diary. Axillary temperature was self-measured using a thermometer and recorded in the same diary twice daily (morning and evening) on Days 1 to 9 and once daily (evening) from Days 10 to 21. In participants taking acetaminophen for antipyretic or analgesic purposes, assessments were postponed until 4 hours after drug administration.

***Definitions of development and worsening of 14 COVID-19 symptoms or fever***

The development (in asymptomatic individuals) or worsening (in patients with mild COVID‑19 symptoms) of any of the 14 COVID-19 symptoms or fever was assessed for each participant using electronic diary data from the start of the study intervention until Day 10. During the severity evaluation at baseline, study participants assessed existing symptoms in the past 30 days (pre-existing symptoms) and the presence or absence of symptom exacerbation due to SARS-CoV-2 infection. To avoid recall bias, the severity of pre-existing symptoms before the onset of SARS-CoV-2 infection was not evaluated.

The development of any of the 14 COVID-19 symptoms or fever in asymptomatic individuals was defined when either of the following criteria (a) or (b) was met:

(a) Shortness of breath (difficulty breathing) and fever:

- A ≥ 1-point worsening of the shortness of breath (difficulty breathing) score from baseline (None at baseline to Mild, Moderate, or Severe; Mild at baseline to Moderate or Severe; Moderate at baseline to Severe), or
- Fever (body temperature < 37.5 °C at baseline rising to ≥ 37.5 °C)

(b) COVID-19 symptoms other than shortness of breath (difficulty breathing):

- Worsening of ≥ 2 symptoms among the following 13 symptoms from baseline at the same time point as defined below, which has been maintained for at least 24 hours:
  - A ≥ 1-point worsening of the stuffy or runny nose, sore throat, cough, low energy or tiredness, feeling hot or feverish, muscle or body aches, headache, chills or shivering, nausea, vomiting, or diarrhea score from baseline (None at baseline to Mild, Moderate, or Severe; Mild at baseline to Moderate or Severe; Moderate at baseline to Severe), or
  - Dysgeusia or anosmia score worsening from 0 (Same as usual) to 1 (Less than usual) or 2 (No sense of smell/taste)

Worsening of any of the 14 COVID-19 symptoms or fever in patients with mild COVID-19 symptoms was defined when either of the following criteria (c) or (d) was met:

(c) Shortness of breath (difficulty breathing) and fever:

- A ≥ 1-point worsening of the shortness of breath (difficulty breathing) score from baseline (None at baseline to Mild, Moderate, or Severe; Mild at baseline to Moderate or Severe; Moderate at baseline to Severe), or
- Fever (body temperature of < 37.5 °C at baseline rising to ≥ 37.5 °C; ≥ 37.5 to < 38.0 °C at baseline rising to ≥ 38.0 °C; ≥ 38.0 to < 38.5 °C at baseline rising to ≥ 38.5 °C)

(d) COVID-19 symptoms other than shortness of breath (difficulty breathing):

- Worsening of ≥ 2 symptoms among the following 13 symptoms from baseline at the same time point as defined below, which had been maintained for at least 24 hours:
  - A ≥ 1-point worsening of the stuffy or runny nose, sore throat, cough, low energy or tiredness, feeling hot or feverish, muscle or body aches, headache, chills or shivering, nausea, vomiting, or diarrhea score from baseline (None at baseline to Mild, Moderate, or Severe; Mild at baseline to Moderate or Severe; Moderate at baseline to Severe), or
  - Dysgeusia or anosmia score worsening from 0 (Same as usual) to 1 (Less than usual) or 2 (No sense of smell/taste)

Participants who rated their symptoms as “Severe” at baseline and those with a baseline body temperature of ≥ 38.5 °C were excluded from the assessment of the development or worsening of COVID-19 symptoms. Moreover, among patients with mild COVID-19 symptoms, those with a score of 2 (No sense of smell/taste) for dysgeusia or anosmia at baseline were excluded from the assessment of the worsening of COVID-19 symptoms.

***Virologic assessments***

Nasopharyngeal swabs were collected from each participant by the investigator or his/her designee on Day 1 (before study intervention) and Days 2 to 6 (Days 3 and 5 were optional), 9, 14, and 21. SARS-CoV-2 viral titer measurements and reverse transcription-polymerase chain reaction tests were centrally performed at ViroClinics (Rotterdam, The Netherlands).

***Sample size rationale and statistical analyses***

The required sample size was calculated for the initially planned primary objective as follows: the proportion of participants who would experience the development or worsening of COVID-19 symptoms was conservatively estimated to be 20% in the placebo group and 8% in the ensitrelvir 125-mg group (risk ratio: 0.4) based on the published literature [2]. Overall, 143 participants were required in each group to detect the differences in the proportion of participants with development or worsening of COVID-19 symptoms between each ensitrelvir group and the placebo group with 80% power using Fisher’s exact test. Assuming a dropout rate of approximately 10% due to a negative reverse transcription-polymerase chain reaction result, enrollment of 480 participants (160 per group) was required.

All statistical comparisons were performed at a two-sided significance level of 0.05. The proportion of participants who experienced the development or worsening of any of the 14 COVID-19 symptoms or fever by Day 10 in each ensitrelvir group was compared with that in the placebo group using the Mantel-Haenszel test. The risk ratio of each ensitrelvir group to the placebo group and its 95% confidence interval (CI) were estimated. Between each ensitrelvir group and the placebo group, the change from baseline in the SARS-CoV-2 viral RNA level was compared using an analysis of covariance (ANCOVA) model that included baseline SARS-CoV-2 viral RNA and SARS-CoV-2 vaccination history (yes or no) as covariates. The proportion of participants with a positive SARS-CoV-2 viral titer was compared using the Mantel-Haenszel test, and the time to the first negative SARS-CoV-2 viral titer was compared using a log-rank test, both of which were stratified by SARS-CoV-2 vaccination history (yes or no). All analyses were performed using SAS version 9.4 (SAS Institute Inc., Cary, NC, USA).

***References***

1. U.S. Food and Drug Administration. Assessing COVID-19-related symptoms in outpatient adult and adolescent subjects in clinical trials of drugs and biological products for COVID-19 prevention or treatment, September 2020. <https://www.fda.gov/media/142143/download/> Accessed April 10, 2024
2. Oran DP, Topol EJ. The proportion of SARS-CoV-2 infections that are asymptomatic: a systematic review. Ann Intern Med 2021;174(5):655-662.

**Table S1** Baseline demographics and clinical characteristics (ITT population).

|  | **Ensitrelvir 125 mg**  **(*n* = 194)** | **Ensitrelvir 250 mg**  **(*n* = 189)** | **Placebo**  **(*n* = 189)** |
| --- | --- | --- | --- |
| Male sex, *n* (%) | 109 (56.2) | 110 (58.2) | 103 (54.5) |
| Age (years), mean (SD) | 37.9 (12.0) | 40.9 (13.4) | 38.6 (13.0) |
| Asian race, *n* (%) | 193 (99.5) | 189 (100.0) | 188 (99.5) |
| COVID-19 vaccination history, *n* (%) | 178 (91.8) | 173 (91.5) | 174 (92.1) |
| SARS-CoV-2 viral RNA level (log_10_ copies/mL), mean (SD)^a^ | 6.43 (1.26) | 6.56 (1.15) | 6.18 (1.48) |
| Participants with any risk factors for severe disease, *n* (%)^b^ | 58 (29.9) | 63 (33.3) | 66 (34.9) |
| Concomitant acetaminophen use, *n* (%) | 45 (23.2) | 56 (29.6) | 44 (23.3) |
| Symptoms, *n* (%) |  |  |  |
| Asymptomatic | 23 (11.9) | 25 (13.2) | 22 (11.6) |
| Mild symptoms^c^ | 171 (88.1) | 164 (86.8) | 167 (88.4) |
| SARS-CoV-2 variant, *n* (%) |  |  |  |
| Delta/21I (Delta) | 1 (0.5) | 0 (0.0) | 0 (0.0) |
| Delta/21J (Delta) | 0 (0.0) | 0 (0.0) | 1 (0.5) |
| BA.1/21K (Omicron) | 53 (27.3) | 47 (24.9) | 50 (26.5) |
| BA.2/21L (Omicron) | 71 (36.6) | 68 (36.0) | 53 (28.0) |
| BA.4/22A (Omicron) | 0 (0.0) | 1 (0.5) | 0 (0.0) |
| BA.5/22B (Omicron) | 5 (2.6) | 8 (4.2) | 3 (1.6) |
| BA.2.12.1/22C (Omicron) | 1 (0.5) | 1 (0.5) | 0 (0.0) |
| Unidentified (recombinant) | 53 (27.3) | 58 (30.7) | 63 (33.3) |
| Not tested | 1 (0.5) | 0 (0.0) | 2 (1.1) |
| No result | 9 (4.6) | 6 (3.2) | 17 (9.0) |

The ITT population comprised all randomized participants who tested positive for SARS‑CoV-2 infection at baseline, as confirmed by an RT-PCR test based on the nasopharyngeal swab sample.

^a^*n* = 194 for the ensitrelvir 125-mg group, *n* = 187 for the ensitrelvir 250-mg group, and *n* = 189 for the placebo group.

^b^Age ≥ 65 years, BMI ≥ 30 kg/m^2^, cancer, cerebrovascular disease, chronic kidney disease, chronic lung disease, chronic liver disease, cystic fibrosis, diabetes mellitus, disabilities, heart conditions, hypertension, dyslipidemia, human immunodeficiency virus, mental health disorders, neurologic conditions, primary immunodeficiencies, smoking, solid organ or hematopoietic cell transplantation, tuberculosis, and the use of corticosteroids or other immunosuppressive medications were considered risk factors for severe disease.

^c^Participants who had only mild symptoms among the 12 symptoms due to COVID-19 within 2 weeks before randomization. Participants assessed the 12 symptoms using a 4-point scale from 0 to 3 (0 = None; 1 = Mild; 2 = Moderate; 3 = Severe).

BMI, body mass index; COVID-19, coronavirus disease 2019; ITT, intention-to-treat; RT‑PCR, reverse transcription-polymerase chain reaction; SARS-CoV-2, severe acute respiratory syndrome coronavirus 2; SD, standard deviation.

**Table S2** Summary of adverse events (safety analysis population).

| **Participants with event, *n* (%)** | **Ensitrelvir**  **125 mg**  **(*n* = 201)** | **Ensitrelvir**  **250 mg**  **(*n* = 202)** | **Placebo**  **(*n* = 201)** |
| --- | --- | --- | --- |
| Any TEAEs | 88 (43.8) | 115 (56.9) | 43 (21.4) |
| TEAEs leading to death | 0 (0.0) | 0 (0.0) | 0 (0.0) |
| Serious TEAEs other than death | 0 (0.0) | 2^a^ (1.0) | 0 (0.0) |
| TEAEs leading to treatment discontinuation | 1 (0.5) | 2 (1.0) | 0 (0.0) |
| Treatment-related adverse events | 47 (23.4) | 75 (37.1) | 14 (7.0) |
| TEAEs occurring in ≥ 2% of participants in either group | | | |
| Headache | 5 (2.5) | 11 (5.4) | 3 (1.5) |
| Nausea | 1 (0.5) | 7 (3.5) | 0 (0.0) |
| Diarrhea | 1 (0.5) | 6 (3.0) | 4 (2.0) |
| High density lipoprotein decreased | 61 (30.3) | 91 (45.0) | 4 (2.0) |
| Blood triglycerides increased | 14 (7.0) | 22 (10.9) | 9 (4.5) |
| Blood bilirubin increased | 7 (3.5) | 15 (7.4) | 0 (0.0) |
| Blood cholesterol decreased | 8 (4.0) | 7 (3.5) | 0 (0.0) |
| Bilirubin conjugated increased | 3 (1.5) | 7 (3.5) | 0 (0.0) |
| Treatment-related adverse events occurring in ≥ 2% of participants in either group | | | |
| High density lipoprotein decreased | 35 (17.4) | 66 (32.7) | 2 (1.0) |
| Blood bilirubin increased | 2 (1.0) | 5 (2.5) | 0 (0.0) |
| Blood triglycerides increased | 0 (0.0) | 5 (2.5) | 1 (0.5) |

The safety analysis population comprised all randomized participants who received at least one dose of ensitrelvir or placebo. Adverse events were coded using the Medical Dictionary for Regulatory Activities version 24.0.

TEAE, treatment-emergent adverse event.

^a^One patient had diverticulitis and the other had nausea. Neither event was treatment related.

**Figure S1** Participant flow.


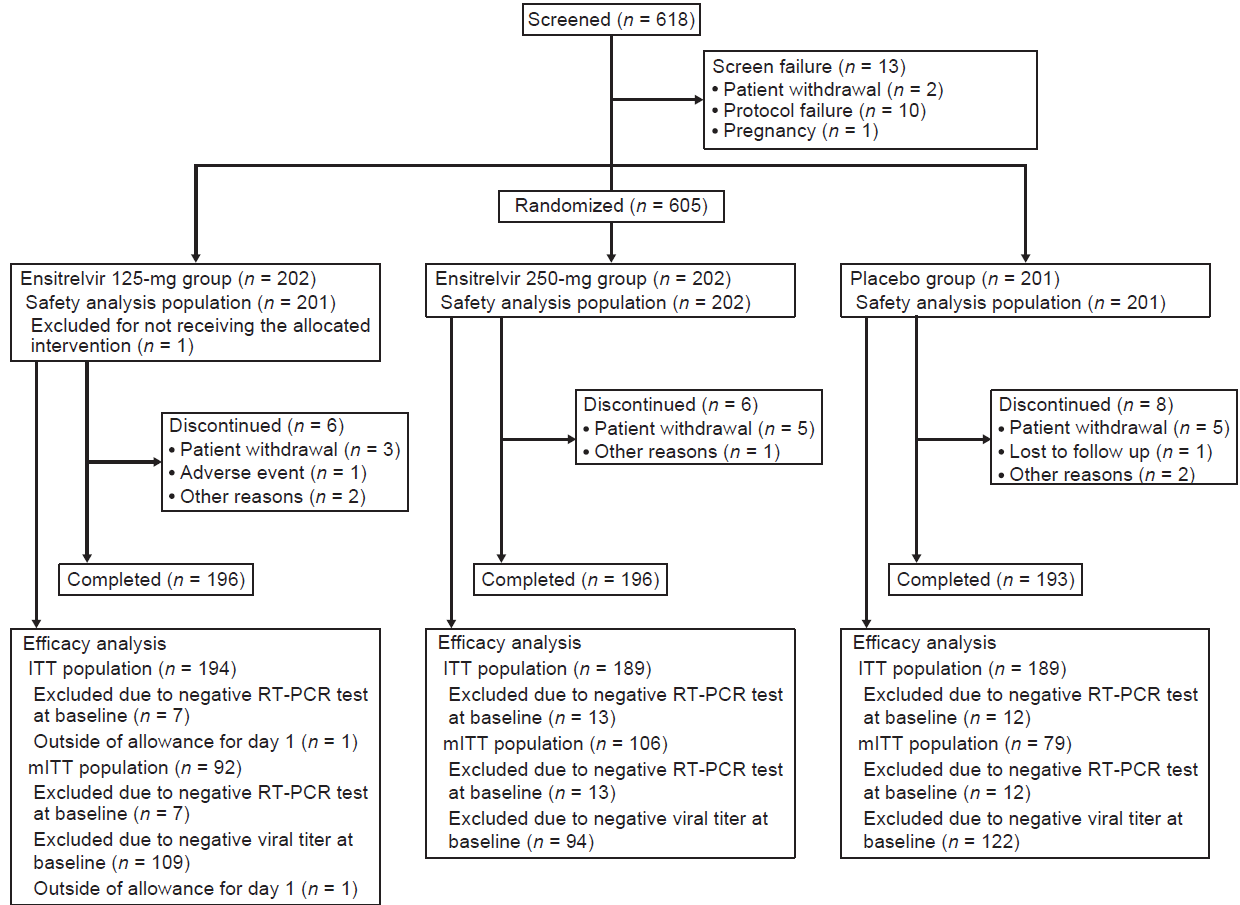


The ITT population comprised all randomized participants who tested positive for SARS‑CoV-2 infection at baseline, as confirmed by an RT-PCR test based on the nasopharyngeal swab sample. The mITT population comprised all randomized participants who tested positive for SARS-CoV-2 infection and had detectable SARS-CoV-2 viral titers at baseline. The safety analysis population comprised all randomized participants who received at least one dose of ensitrelvir or placebo. Some patients were excluded from the analysis for more than one reason.

ITT, intention-to-treat; mITT, modified intention-to-treat; RT-PCR, reverse transcription‑polymerase chain reaction; SARS-CoV-2, severe acute respiratory syndrome coronavirus 2.

**Figure S2** (A) Observed values and (B) change from baseline in SARS‑CoV‑2 RNA levels (ITT population).


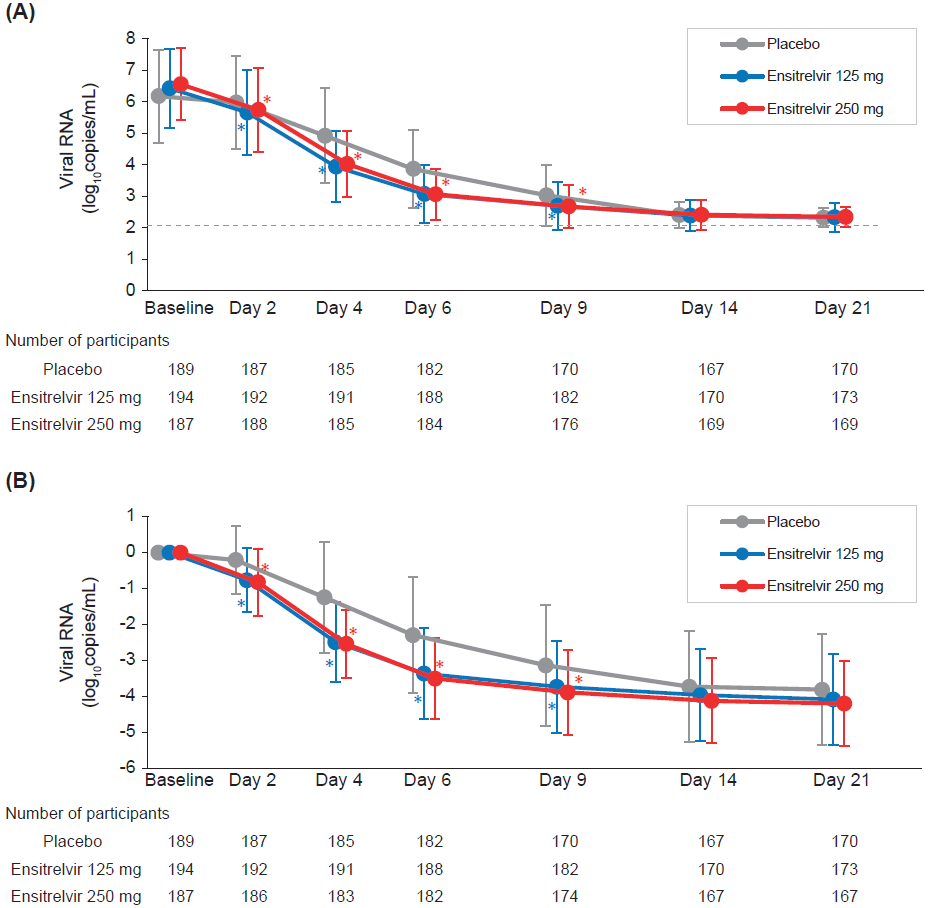


The ITT population comprised all randomized participants who tested positive for SARS‑CoV-2 infection at baseline, as confirmed by an RT-PCR test based on the nasopharyngeal swab sample. Data are presented as mean ± SD. The dotted line represents the lower limit of quantification for SARS-CoV-2 RNA level (2.08 log_10_ copies/mL). Analyses were performed using an ANCOVA model that included baseline SARS-CoV-2 viral RNA and SARS-CoV-2 vaccination history (yes or no) as covariates. **p* < 0.05 vs. placebo.

ANCOVA, analysis of covariance; ITT, intention-to-treat; RNA, ribonucleic acid; RT-PCR, reverse transcription-polymerase chain reaction; SARS-CoV-2, severe acute respiratory syndrome coronavirus 2; SD, standard deviation.

**Figure S3** Kaplan-Meier plot for the time to first negative infectious SARS‑CoV-2 viral titer (infectious viral clearance) (mITT population).


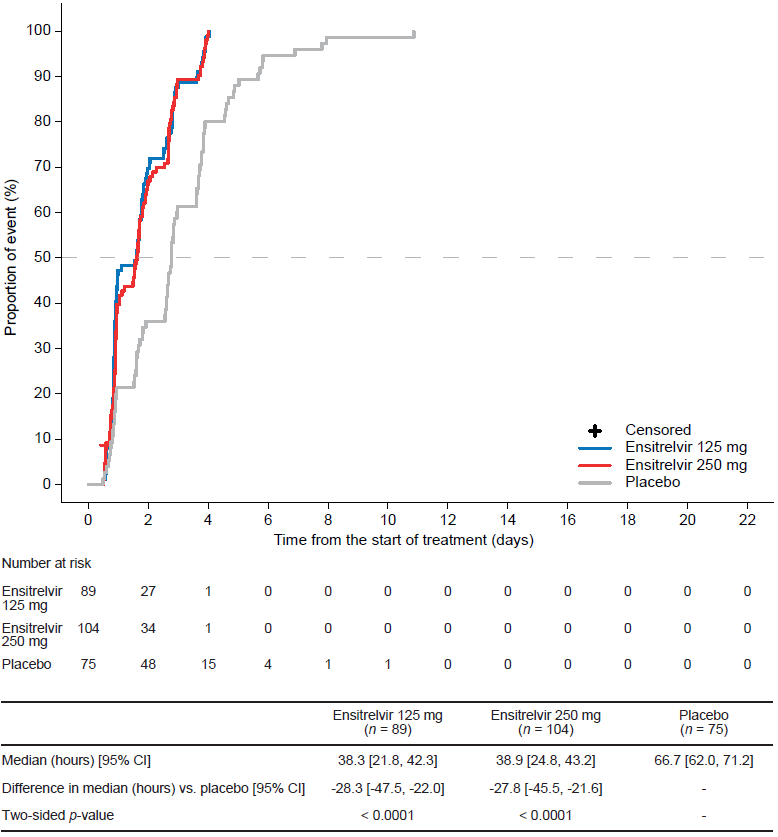


The mITT population comprised all randomized participants who tested positive for SARS‑CoV-2 infection and had detectable SARS-CoV-2 viral titers at baseline. The time to the first negative SARS-CoV-2 viral titer for each ensitrelvir group was compared with that of the placebo group by using a log-rank test stratified by SARS-CoV-2 vaccination history (yes or no).

CI, confidence interval; mITT, modified intention-to-treat; SARS-CoV-2, severe acute respiratory syndrome coronavirus 2.
